# Cholinergic REST-G9a gene repression through HMGB1-TLR4 neuroimmune signaling regulates basal forebrain cholinergic neuron phenotype

**DOI:** 10.3389/fnmol.2022.992627

**Published:** 2022-08-22

**Authors:** Fulton T. Crews, Ryan P. Vetreno

**Affiliations:** ^1^Bowles Center for Alcohol Studies, School of Medicine, University of North Carolina at Chapel Hill, Chapel Hill, NC, United States; ^2^Department of Psychiatry, School of Medicine, University of North Carolina at Chapel Hill, Chapel Hill, NC, United States

**Keywords:** epigenetic, basal forebrain cholinergic neuron, choline acetyltransferase, neuroimmune, acetylcholine, methyltransferase, neuronal plasticity

## Abstract

Lipopolysaccharide (LPS) and high-mobility group box 1 (HMGB1) are Toll-like receptor (TLR4) agonists that activate proinflammatory neuroimmune signaling linked to loss of basal forebrain cholinergic neurons (BFCNs) and cognitive deficits. Loss of choline acetyltransferase immunoreactive (ChAT + IR) BFCNs is generally interpreted as cell death, but recent *in vivo* studies find anti-inflammatory interventions restore adolescent ethanol exposure-induced persistent loss of adult ChAT + IR neurons and cognitive deficits, suggesting proinflammatory signaling-induced reversible gene repression of ChAT in BFCNs. Using an *ex vivo* Wistar rat basal forebrain slice culture (FSC) model to investigate TLR4 involvement in repression of the BFCN phenotype, we report that direct TLR4 activation with LPS decreases expression of multiple BFCN markers in the absence of observable neuronal loss or cell death. Inhibition of HMGB1 blunts while inhibition of TLR4 blocks the LPS-induced loss of ChAT + IR neurons. TLR4 activation induces the transcriptional repressor RE1-silencing transcription factor (REST) and the methyltransferase G9a while increasing repressive histone 3 lysine 9 dimethylation and REST occupancy at cholinergic gene promoters. G9a inhibitors both prevent and reverse the LPS-induced loss of ChAT + IR whereas siRNA inhibition of REST blocks the LPS-induced loss of ChAT + IR BFCNs. These data suggest *in vivo* HMGB1-TLR4 signaling in BFCNs leads to a reversible loss of the cholinergic neuron phenotype through epigenetic gene repressive mechanisms.

## Introduction

Basal forebrain cholinergic neurons (BFCNs) provide extensive innervation to the hippocampus and cortex (Mesulam et al., 1983), and are critically involved in cognition and brain function (Blake and Boccia, 2018). Loss of BFCNs and somal shrinkage of the remaining BFCNs are features of several neurodegenerative disorders, including Alzheimer’s disease (AD) and alcohol use disorder (AUD) (Lehericy et al., 1993; Vetreno et al., 2014), and likely contribute to the cognitive dysfunction associated with these disorders (Vogels et al., 1990; De Rosa et al., 2004; Mufson et al., 2008). BFCN loss in AD and AUD (Lehericy et al., 1993; Vetreno et al., 2014) is accompanied by increases of Toll-like receptor 4 (TLR4) and the endogenous TLR ligand HMGB1 (Crews et al., 2013; Vetreno et al., 2013; Paudel et al., 2020). HMGB1 binds to and activates TLR4 and other receptors, leading to nuclear translocation of NFκB, contributing to complex proinflammatory signaling (Mayfield et al., 2013). In a rodent adolescent intermittent ethanol (AIE) model of human adolescent binge drinking, alcohol decreases BFCNs and increases forebrain expression of HMGB1, TLR4, and downstream activated pNFκB p65 that persists into adulthood (Crews et al., 2021). *In vivo*, BFCNs express TLR4 and activated pNFκB p65, suggesting that these neurons can respond to TLR4 agonists and other neuroimmune signals (Crews et al., 2021). Systemic administration of the TLR4 ligand LPS induces lasting upregulation of NFκB target genes (e.g., TNFα, CCL2 [MCP-1], IL-1β) (Qin et al., 2007, 2008) in brain and decreases BFCNs (Vetreno and Crews, 2018). However, systemic LPS exposure also induces peripheral cytokines (e.g., TNFα) that can drive neuroinflammation and progressive neurodegeneration (Qin et al., 2007), confounding interpretation of direct HMGB1-TLR4 neuroimmune involvement in the loss of BFCNs.

Cholinergic neurons develop early, but continue to mature through youth and adolescence into adult BFCNs that express cholinergic lineage transcription factors and other markers, such as ChAT, VAChT (SLC18A3), the choline transporter (ChT, SLC5A7), and the NGF receptor tropomyosin receptor kinase A (TrkA). Loss of ChAT + IR and other cholinergic neuron markers is generally considered analogous to cell death of BFCNs; however, fimbria-fornix lesion-induced loss of ChAT + IR BFCNs can be restored post-lesion by intraventricular infusions of NGF (Hagg et al., 1989). Recent studies find AIE exposure causes a persistent increase in adult basal forebrain HMGB1-TLR4 neuroimmune gene expression and a long-lasting loss of ChAT + IR BFCNs that is prevented and reversed by anti-inflammatory drug treatment and exercise (Vetreno et al., 2019; Crews et al., 2021). Loss of BFCNs markers *in vivo* is accompanied by increased occupancy of the transcriptional repressive marker histone 3 lysine 9 dimethylation (H3K9me2) at *Chat* and *Trka* gene promoters in the basal forebrain (Wang et al., 2008; Vetreno et al., 2019) that can be reversed (Vetreno et al., 2019; Crews et al., 2021). Together, these data suggest that proinflammatory signaling involving HMGB1-TLR4 signaling may reduce BFCNs through repressive epigenetic gene silencing mechanisms that can be reversed.

Emerging studies suggest neuroimmune induction can elicit chromatin remodeling and gene transcription regulation in brain through epigenetic modifications (Montesinos et al., 2016; Chastain and Sarkar, 2017; Wolstenholme et al., 2017; Nestler and Luscher, 2019). Epigenetic transcriptional regulation involves histone acetylation and methylation, which can enhance or repress gene transcription without changing the underlying DNA sequence, resulting in a specific phenotype (Kouzarides, 2007; Berger et al., 2009; Fernandes et al., 2017). In the current study, we used an *ex vivo* basal forebrain slice culture (FSC) model, which maintains the cellular milieu conducive for BFCN development, to overcome *in vivo* systemic confounds to test the hypothesis that neuroimmune activation causes epigenetic repression of the BFCN phenotype. Using the FSC model, we directly link HMGB1-TLR4 neuroimmune signaling with induction of REST-G9a-H3K9me2 transcriptional silencing markers on cholinergic phenotype genes that culminates in epigenetic repression of ChAT and other cholinergic genes. These data reveal that HMGB1-TLR4 neuroimmune activation induces a novel neuroplastic process involving epigenetic gene repression, resulting in the loss of the BFCN phenotype.

## Materials and methods

### Animals

For *in vivo* LPS studies, Wistar rats were bred and reared at the University of North Carolina at Chapel Hill. On the day following birth (P1), litters remained with their dams in standard clear plastic tubs with shavings until the time of weaning on P21. Subjects were housed in a temperature- (20°C) and humidity-controlled vivarium on a 12/12 h light/dark cycle (light onset at 7:00 A.M.), and provided *ad libitum* access to food and water. This study was conducted in an AAALAC-accredited facility in strict accordance with NIH regulations for the care and use of animals in research. Experimental procedures reported in this study are approved by the Institutional Animal Care and Use Committee of the University of North Carolina at Chapel Hill.

### *In vivo* lipopolysaccharide treatment

**FIGURE 1:**
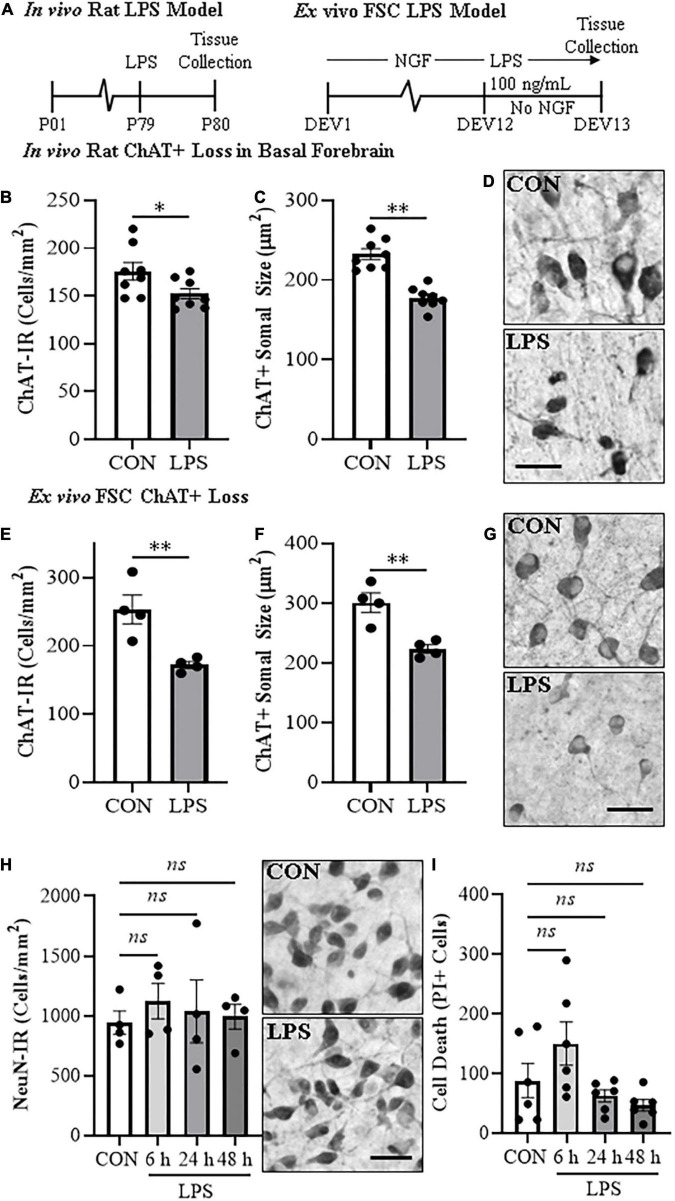
*In vivo* and *ex vivo* reductions of ChAT + IR neurons and ChAT+somal shrinkage in the basal forebrain. **(A)** Schematic depicting experimental design for *in vivo* and *ex vivo* lipopolysaccharide (LPS) experiments. For the *in vivo* study, naïve Wistar rats received a single dose of LPS (1.0 mg/kg, i.p.) on postnatal day (P) 79 and tissue collected on P80. For the *ex vivo* studies, basal forebrain slice cultures (FSC) were treated with NGF (100 μg/mL) until day *ex vivo* (DEV) 12. On DEV12, FSCs were treated with LPS (100 ng/mL) or vehicle for 24 h in the absence of NGF until tissue collection on DEV13. **(B)** Modified unbiased stereology revealed that systemic administration of the proinflammatory TLR4 ligand LPS caused a 13% (±3%) reduction of ChAT + IR cholinergic neurons in the basal forebrain 24 h post-LPS treatment relative to vehicle-treated CONs (*n* = 8/group, two-tailed *t*-test). **(C)** Analysis of ChAT+ neuron somal size 24 h post-LPS treatment revealed a 24% (±2%) reduction in somal size of the remaining ChAT+ neurons relative to CONs (*n* = 8/group, two-tailed *t*-test). **(D)** Representative photomicrographs of ChAT + IR neurons in the basal forebrain of CON- and LPS-treated subjects. Note somal shrinkage of the remaining ChAT+ cholinergic neurons following LPS treatment relative to CONs. **(E)** Modified unbiased stereological assessment revealed that direct application of LPS to FSC media caused a 32% (±2%) reduction of ChAT + IR neurons relative to vehicle-treated CONs (*n* = 4 wells/group, two-tailed *t*-test). **(F)** Analysis of somal size revealed a 26% (±2%) reduction in somal size of the remaining ChAT+ neurons in LPS-treated FSC relative to vehicle-treated CONs (*n* = 4 wells/group, two-tailed *t*-test). **(G)** Representative photomicrographs of ChAT + IR neurons from CON- and LPS-treated FSCs. Note somal shrinkage of the remaining ChAT+ cholinergic neurons following LPS treatment relative to CONs. **(H)** Modified unbiased stereological assessment revealed that direct application of LPS to FSC media did not affect expression of NeuN + IR neurons. Time-course assessment (i.e., 6, 24, and 48 h) of NeuN + IR revealed that LPS treatment did not affect neuronal marker expression across time points, relative to CON at 24 h (one-way ANOVA with Tukey’s HSD). Representative photomicrographs of NeuN + IR neurons from CON- and LPS-treated FSCs at the 24 h time point. **(I)** Assessment of propidium iodide (PI) fluorescent immunoreactive cells revealed that direct application of LPS to FSC media did not increase PI + IR cells. Time-course assessment (i.e., 6, 24, and 48 h) of PI + IR revealed that LPS treatment did not increase cell death across time points, relative to CON at 24 h (one-way ANOVA with Tukey’s HSD). Scale bar = 50 μm. Data are presented as mean ± SEM. *p < 0.05, ^**^p < 0.01.

To determine if systemic administration of the proinflammatory TLR4 ligand LPS would alter ChAT + IR BFCNs *in vivo*, rats (*n* = 8/group) received either a single injection of LPS (1.0 mg/kg, i.p. in sterile 0.9% saline; *Escherichia coli*, serotype 0111:B4; Sigma-Aldrich, Cat. #L3024) or a comparable volume of vehicle on P79. Subjects were sacrificed on P80 and brain tissue collected for analysis (Figure 1A).

### *In vivo* perfusion and brain tissue preparation

Subjects were anesthetized with a lethal dose of sodium pentobarbital (100 mg/kg, i.p.) and transcardially perfused with 0.1 M PBS followed by 4.0% PFA. Brains were excised and post-fixed in 4.0% PFA for 24 h at 4°C followed by 4 days fixation in 30% sucrose solution. Coronal sections were cut (40 μm) on a sliding microtome (MICROM HM450; Thermo Scientific, Austin, TX, United States) and sections sequentially collected into well plates and stored at −20°C in a cryoprotectant solution (30% glycol/30% ethylene glycol in PBS). Free-floating basal forebrain tissue samples [every 6th section; approximate Bregma: 1.60–0.20 mm based on the atlas of Paxinos and Watson (1998)] were used.

### *Ex vivo* organotypic forebrain slice culture model

For *ex vivo* slice culture studies, P8 Wistar rat pups from Charles River Laboratory (Wilmington, MA, United States) were used based on prior studies (Humpel, 2015). FSCs were prepared as described previously (Weis et al., 2001; Zou and Crews, 2012, 2014; Zou et al., 2012) with slight modification. Briefly, neonate (P8) rats were decapitated, brain excised, and coronal slices sectioned with a McIlwain mechanical tissue chopper at a thickness of 375 μm. Basal forebrain slices were dissected in Gey’s buffer (Sigma-Aldrich, St. Louis, MO, United States) and two slices containing the entire basal forebrain from each animal were placed onto a 30 mm diameter membrane tissue insert. Individual tissue inserts were cultured in six well well-plates with medium containing 75% glutamate-free MEM with 25 mM HEPES and Hank’s salts supplemented with 25% heat-inactivated horse serum, 5.5 g/L glucose, 2.0 mM L-glutamine, and 100 μg/mL nerve growth factor (NGF; Sigma-Aldrich, Cat. #N2513) in a humidified 5.0% CO_2_ incubator at 36.5°C. FSCs were maintained in culture for 12 days with NGF, which is critical for establishing BFCNs (Supplementary Figures 1A–C). It is well-established that slices become thinner during 12 days of incubation (Weis et al., 2001; Ehrlich et al., 2012), which is a sign of healthy cultures, and slices that did not become thinner, or wells containing dead or contaminated slices were immediately removed from experiments. Basal forebrain slices in culture for 12 days *ex vivo* (DEV) were used for experiments, and drug treatments performed in the absence of NGF as FSCs survive up to an additional 12 DEV in the absence of continued NGF (Weis et al., 2001; Supplementary Figure 1D).

### *Ex vivo* forebrain slice culture lipopolysaccharide and drug treatments

For LPS experiments, FSCs were treated with the TLR4 ligand LPS (100 ng/mL; *E. coli*, serotype 0111:B4; Sigma-Aldrich, Cat. #L3024) in media for 6–48 h. All drug treatments were performed in NGF-free medium after 12 DEV. In some experiments, disulfide HMGB1 [HMGBiotech, Cat. #HM-121; 0.1 μg/mL [Venereau et al., 2012]), glycyrrhizin (Sigma-Aldrich, Cat. #G2137; 100 μM (Zou and Crews, 2014)], LPS-RS [InvivoGen, San Diego, CA, United States, Cat. #tlrl-prslps; 100 ng/mL (Zou and Crews, 2014)], or G9a inhibitors (BIX-01294 [Selleckchem, Houston, TX, United States, Cat. #S8006; 5.0 μM], UNC0642 [Santa Cruz, Dallas, TX, United States, Cat. #sc397059; 1.0 μM]) were used as described in the “Results” section (*n* = 3–6 wells/group per experiment). For knockdown of REST, a Silencer Select REST siRNA cocktail and Silencer Select scrambled negative control siRNA (Ambion, Grand Island, NY, United States, Cat. #s136124 [100 nM], #s136125 [100 nM]; #s136126 [100 nM]; negative control siRNA, Cat. #4390843) was used based on the protocol for transfection as previously described (Zou et al., 2012; Zou and Crews, 2014). Briefly, the transfection mixture was added to the media at a final concentration of 100 nM of each siRNA + 8.0 μL Lipofectamine 2000 (Invitrogen, Carlsbad, CA, United States) in Gibco Opti-MEM Reduced Serum Medium to a total volume of 1.1 mL (550 μL on top of slices and 550 μL at the bottom of the slice culture). Negative controls were treated with the same medium containing the scrambled negative control siRNA. After transfection for 24 h, siRNA-containing medium was replaced with regular medium without NGF and the slices cultured for 24 h in the absence or presence of LPS (100 ng/mL). At the end of all experiments, slices were collected from tissue inserts for analysis. Two brain slices containing the basal forebrain from each animal were cultured in each well with multiple wells used in each experiment as described in the figure captions.

For immunohistochemistry (IHC) studies, membrane tissue inserts were collected from each well (*n* = 3–6 wells/group), fixed in a solution of 4.0% PFA and 5.0% sucrose in 0.1 M PBS (pH 7.4) for 24 h, and stored in 0.1 M PBS. For RNA (*n* = 4–6 wells/group) and DNA (*n* = 6–10 wells/group) extraction, membrane tissue inserts containing FSCs were rinsed in cold 0.1 M PBS, removed from membrane tissue inserts, and stored at −80°C.

### Assessment of neuronal cell death

Uptake of the fluorescent exclusion dye propidium iodide (PI) was used for determination of neuronal cell death as previously described (Rudolph et al., 1997; Coleman et al., 2017). Briefly, PI was added to the culture medium at the beginning of LPS treatment at a concentration of 5.0 μg/mL and PI fluorescent images captured at 6, 24, and 48 h relative to CONs at 24 h. PI intercalates into the DNA of non-viable cells but cannot enter viable cells as it is excluded by the plasma membrane providing a measure of cell death (Rudolph et al., 1997). This method is well characterized for accurately measuring neuronal cell death in organotypic slice culture (Zou and Crews, 2005). PI fluorescent immunoreactive cells were imaged using AxioVision 3.1 software and quantified by an experimenter blind to condition using ImageJ software.

### Immunohistochemistry

Free-floating basal forebrain tissue was washed in 0.1 M PBS, incubated in 0.6% H_2_O_2_ to inhibit endogenous peroxidases, and blocked with normal serum (MP Biomedicals, Solon, OH, United States). Sections were incubated in a primary antibody solution containing blocking solution and goat anti-ChAT (Millipore, Temecula, CA, United States, Cat. #AB144P, RRID:AB_2079751), mouse anti-TLR4 (Abcam, Cat. #ab22048, RRID:AB_446735), or mouse anti-NeuN (Millipore, Cat. #MAB377, RRID:AB_2298772) for 24 h at 4°C. Sections were washed with PBS, incubated for 1 h in a biotinylated secondary antibody (Vector Laboratories, Burlingame, CA, United States), and incubated for 1 h in ABC solution (Vectastain ABC Kit; Vector Laboratories). The chromogen nickel-enhanced DAB (Sigma-Aldrich) was used to visualize immunoreactivity. Tissue was mounted onto slides, dehydrated, and cover slipped. Negative controls for non-specific binding were conducted on separate sections employing the above-mentioned procedures, omitting the primary antibody.

### Microscopic quantification and image analysis

Across experiments, BioQuant Nova Advanced Image Analysis software (R&M Biometric, Nashville, TN, United States) was used for image capture and quantification of IHC. Representative images were captured using an Olympus BX50 microscope and Sony DXC-390 video camera linked to a computer. For each measure, the microscope, camera, and software were background-corrected and normalized to preset light levels to ensure fidelity of data acquisition. Microscopic quantification, which was performed by experimenters blind to treatment conditions, was conducted in the medial septum and diagonal band (Vetreno et al., 2014). A modified unbiased stereological quantification method was used to quantify ChAT + IR, TLR4 + IR, and NeuN + IR cells in the basal forebrain. We previously reported that comparison of traditional unbiased stereological methodology with our modified unbiased stereological approach yielded nearly identical values for heterogeneously distributed cell populations (Crews et al., 2004). The outlined regions of interest were determined and data expressed as cells/mm^2^. ChAT + IR somal size was assessed using BioQuant Nova Advanced Image Analysis software (R&M Biometric).

### Fluorescent immunohistochemistry and microscopy

Free-floating FSC sections were processed similar to previously reported methods (Vetreno and Crews, 2018; Vetreno et al., 2019). To assess cholinergic neuron marker colocalization, sections were incubated for 48 h at 4°C in a primary antibody cocktail containing goat anti-ChAT (Millipore), rabbit anti-TrkA (Millipore, Cat. #06-574, RRID:AB_11213262), and mouse anti-nerve growth factor receptor (NGFR; Millipore, Cat. #MAB365, RRID:AB_2152788). To assess ChAT colocalization with phosphorylated (activated) NF-κB p65, FSC sections were incubated for 48 h at 4°C in a primary antibody cocktail containing goat anti-ChAT (Millipore) and rabbit anti-pNF-κB p65 (phospho S536; Abcam, Cat. #ab86299, RRID:AB_1925243). Sections were then washed in TBS and incubated for 2 h at room temperature in the secondary antibody cocktail (Invitrogen; rabbit Alexa Fluor 594 [Cat. #A21207, RRID:AB_141637], mouse Alexa Fluor 488 [Cat. #A21202, RRID:AB_141607], and goat Alexa Fluor 350 [Cat. #A21081, RRID:AB_2535738]). Tissue was mounted onto slides and cover slipped using Prolong Gold Anti-Fade mounting media (Life Technologies, Grand Island, NY, United States). Immunofluorescent images were obtained using a DS-RiZ scope (Nikon Inc., Melville, NY, United States), and colocalization and pNF-κB p65 + IR cells quantified using NIS Elements AR46 (Nikon Inc.).

### ELISA

At the conclusion of LPS treatment for 24 h in the FSC, media was collected and used for detection of HMGB1 release. HMGB1 ELISA was performed according to the manufacturer’s protocol (IBL International, Hamburg, Germany [Cat. #ST51011]).

### RNA extraction and reverse transcription PCR

Across experiments, total mRNA was extracted from FSC samples by homogenization in TRI reagent (Sigma-Aldrich) following the single-step method of RNA isolation (Chomczynski and Sacchi, 2006) as previously described (Vetreno and Crews, 2018; Vetreno et al., 2018; Liu et al., 2020). Briefly, total RNA was extracted from individual FSC wells by homogenization in TRI reagent (Sigma-Aldrich). RNA quality and concentration was determined using a NanoDrop 1000 (Thermo Fisher Scientific, Austin, TX, United States). Total RNA was reverse transcribed as previously described (Vetreno et al., 2018). RTPCR reactions were run on a Bio-Rad CFX system (Bio-Rad, Hercules, CA, United States) and primer sequences are presented in Table 1. SYBER Green PCR Master Mix (Life Technologies, Carlsbad, CA, United States) was used for RTPCR. RTPCR was run with an initial activation for 10 min at 95°C, followed by 40 cycles of denaturation (95°C, 15 s), annealing/extension (57–58°C, 1 min), and melt curve. Differences in primer extension between groups are expressed as cycle time (Ct) values normalized to a housekeeping gene (i.e., β *actin* or *18S*), and relative differences between groups calculated and expressed as the percent difference relative to controls.

**TABLE 1:** List of primer sequences for RTPCR analysis.

Primer	Forward	Reverse
*Ache*	GACTGCCTTTATCTTAA TGTG	CGGCTGATGAGAGATT CATTG
*Acly*	CAGCAGGACAGCGTC TTTTTC	GGGATCTTGGACTTG GGACT
*Ccl2*	CTGGGCCTGTTGTTCA CAGTTGC	CTACAGAAGTGCTTG AGGTGGTTG
*Cd11b*	CTGGTACATCGAGAC TTCTC	TTGGTCTCTGTCTGAG CCTT
*Cdyl*	GCTGTTAATGGAAAA GGTACATCTC	CTCACACTGAAACGC AACCG
*Chat*	GCCCAACCAAGCCA AGCAAT	AAATGTCTTTGCGG GTGCCG
*Cht*	CATCACAGAACCTCACT CACAC	GCAAGAGGCTGAAACA TTTGGG
*G9a*	CTCCGGTCCCTTGT CTCC	CTATGAGAGGTGTCC CCCAA
*Gbx1*	GCCAAGTGGAAGCGAA TCAAG	CTCTGGCCGAATACTC ATGC
*Gbx2*	CACTTGAGGGAACCCG TGTC	AAACCGCAGTGTTCT TGGGT
*Gfap*	AATGACTATCGCCGCC AACT	CGAGTGCCTCCTGGTA ACTC
*Glt1*	GGACTGGCTGCTGG ATAGA	ATGGTAAGAATGGATGC AGGGG
*Gs*	CGGGTGTACTTGTGGT GAGG	GGGTGAACTCCCCTT CCCTA
*Iba1*	GGCAATGGAGATATCG ATA	AGAATCATTCTCAAGA TGGC
*Il1*β	GAAACAGCAATGGTCG GGAC	AAGACACGGGTTCCA TGGTG
*Il6*	CTGGTCTTCTGGAGTT CCGTT	GGTCTTGGTCCTTAG CCACTC
*Isl1*	TCCCTATGTGTTGGT TGCGG	AGCAGGTACAGCTT TCGTCC
*Ldb1*	CATGCGAGTCTTTGT GCGG	GTGGGTACATGGGAGT TGGG
*Lhx8*	GCCTTGGTAGAGGAGAA GGTC	TGGCTGGCTTTGGATGA TTGA
*Neun*	CCCACCACTCTCTT GTCCGT	GGGCCGATGGTATGAT GGTAG
*Ngfr*	GCTGCTGATTCTAGG GATGTC	CAGTCTCCTCGTCC TGGTAGT
*Rest*	ACTACACGGCACACC TGAAG	GAGGTTTAGGCCCGT TGTGA
*S100B*	GAGAGAGGGTGACAA GCACAA	GGCCATAAACTCCT GGAAGTC
*Tnf*α	ATGTGGAACTGGCA GAGGAG	ACGAGCAGGAATGAG AAGAAG
*Trka*	CCATATCAAGCGCCAG GACA	GCAGTTTTGCATCA GGTCCG
*Vacht*	AGGCCACATCGTTCACTCTC	GGCGGTTCATCAAGCAACAC
β *actin*	CTACAATGAGCTGCG TGTGGC	CAGGTCCAGACGCAGGA TGGC
*18s*	CGGGGAATCAGGGTT CGATT	TCGGGAGTGGGTAAT TTGCG

### Chromatin immunoprecipitation

The procedure used was similar to methods reported previously (Kyzar et al., 2017; Vetreno et al., 2019; Crews et al., 2021). Briefly, FSCs were homogenized, fixed in 1.0% methanol-free formaldehyde, quenched in 1.0 M glycine, lysed with lysis buffer (1.0% [v/v] SDS, 10 mM EDTA, 50 mM Tris-HCl [pH 8.0]), and chromatin sheared to fragments of <1,000 bp on a Covaris ME220. Input DNA fractions were removed from the sheared chromatin to be processed separately and the remaining sheared chromatin was incubated overnight at 4°C with an antibody against H3K9me2 (Abcam, Cat. #ab1220, RRID:AB_449854) or REST (Millipore, Cat. #17-641, RRID:AB_1977463). Protein A Dynabeads (Thermo Fisher Scientific, Austin, TX, United States) were added and rotated at 4°C for 1 h followed by five washes in ChIP wash buffer. Both immunoprecipitated DNA and input DNA were then eluted in 10% (w/v) Chelex by boiling at 95°C for 10 min followed by centrifugation. The resulting DNA was quantified using qPCR with SSOAdvanced Universal SYBR Green Supermix (Bio-Rad, Berkeley, CA, United States) using primers for promoter and promoter CpG islands at *Chat*, *Trka*, and *Lhx8* genes (Table 2). The ΔΔCt method was used to determine fold change relative to control and was normalized to the Input DNA fraction.

**TABLE 2:** List of primer sequences for ChIP analysis.

Primer	Forward	Reverse	Chromosome, sequence region
*Chat* promoter	ACTTGATTGCTGCCTCTCTC	GGGATGGTGGAAGATACAGAAG	Chr 16, 7717766–7717785
*Chat* CpG promoter	TGCATCTGGAGCTCAAATCGT	GGGGATAGTGGTGACGTTGT	Chr 16, 7717354–7717374
*Trka* promoter	CCTCACCGTGCACTTTACCT	AGGGTCTGGAGAGCGTACAT	Chr 2, 173254387–173254406
*Trka* CpG promoter	TCAAGCAAGGCTCCGAACAG	CACAGGGTGGCGCTAGAAG	Chr 2, 173254107–173254126
*Lhx8* promoter	ATCGGAGGCGGTGTATGTTC	TGGGCCTGGTTCGGATTAAG	Chr 2, 243270737–243270756

### Statistical analysis

Statistical analysis was performed using GraphPad Prism 8 (San Diego, CA, United States). Two-tailed Student’s *t*-tests were used to assess RTPCR, IHC, and ChIP data unless otherwise reported. Levene’s Test for Equality of Variances was performed for each analysis. When reported in the Section “Results,” Welch’s *t*-tests were used to assess data with unequal variances. All time course and dose response data was assessed using one-way ANOVA with Tukey’s HSD. Blockade studies were analyzed using 2 × 2 ANOVAs with *post hoc* analyses performed using Tukey’s HSD where appropriate. If significant interactions were not observed, follow-up *t*-tests were performed to determine blockade. All values are reported as mean ± SEM, and significance defined as *p* < 0.05.

## Results

### Lipopolysaccharide -TLR4 activation contributes to loss of basal forebrain cholinergic neurons and somal shrinkages of ChAT + IR neurons

Loss of BFCNs and proinflammatory TLR4 neuroimmune induction are features of several neurodegenerative disorders, including AD and AUD (Lehericy et al., 1993; Crews et al., 2013; Vetreno et al., 2014; Paudel et al., 2020). To investigate mechanisms regulating BFCN responses to proinflammatory signaling, we assessed whether TLR4 neuroimmune activation with LPS contributes to the loss of BFCNs *in vivo* and in *ex vivo* FSC. Development of BFCNs in the FSC model is dependent on NGF, with cultured cholinergic neurons co-expressing ChAT, TrkA and the NGF receptor, similar to development of BFCNs *in vivo* (Supplementary Figure 1E) (Vetreno and Crews, 2018; Vetreno et al., 2019). Systemic treatment of Wistar rats with LPS (1.0 mg/kg, 24 h) decreased ChAT + IR cells (*t*[14] = 2.2, *p* < 0.05) and reduced somal size of the remaining ChAT+ neurons (*t*[14] = 6.6, *p* < 0.01), relative to CONs (Figures 1B–D), similar to previous *in vivo* studies finding persistent ChAT loss following LPS lasting at least 10 days (Vetreno and Crews, 2018). *Ex vivo* FSC LPS (100 ng/mL, 24 h [Figure 1A]) treatment caused a 32% (±2%) reduction of ChAT + IR neurons (*t*[6] = 3.8, *p* < 0.01) relative to vehicle-treated FSCs. Similar to the *in vivo* LPS-induced somal shrinkage, direct LPS-TLR4 activation caused a 26% (±2%) reduction in somal size of the remaining ChAT + IR cholinergic neurons (*t*[6] = 4.4, *p* < 0.01) relative to vehicle-treated FSCs (Figures 1E–G). Although loss and shrinkage of BFCNs could be interpreted as cell death and autophagy, no changes in NeuN + IR neurons or the cell death marker PI were found across a 6–48 h time course (Figures 1H,I). These data suggest that *ex vivo* LPS reduces ChAT + IR BFCNs without causing observable cell death or loss of NeuN + neurons. LPS treatment of BFCNs within the FSC model reveals that direct TLR4 activation can decrease expression of ChAT + IR cholinergic neurons independent of systemic responses and loss of neurons.

**FIGURE 2:**
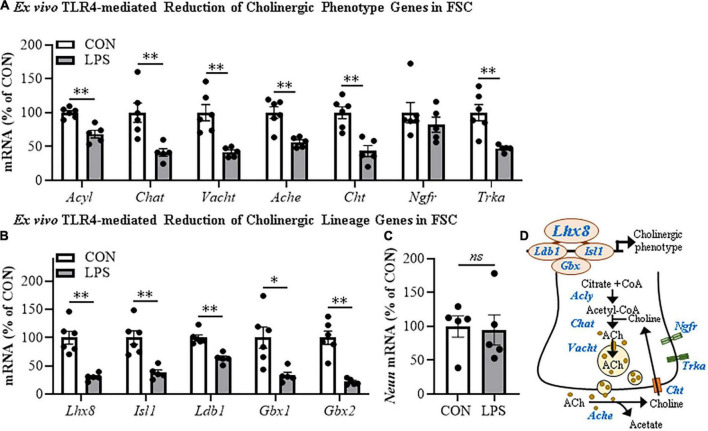
Direct TLR4 activation with LPS reduces cholinergic neuron gene expression in the FSC model. Reverse transcription PCR (RTPCR) analysis revealed that direct application of LPS (100 ng/mL; 24 h) to basal FSC media decreased mRNA expression of **(A)** cholinergic phenotype (*Acyl* [31% (±6%)], *Chat* [59% (±6%)], *Vacht* [58% (±3%)], *Ache* [43% (±3%)], *Cht* [57% (±8%)], *Trka* [53% (±2%)]) and **(B)** lineage genes (*Ldb1* [37% (±4%)], *Isl1* [62% (±5%)], *Lhx8* [70% (±3%)], *Gbx1* [67% (±6%)], *Gbx2* [78% (±2%)]) relative to vehicle-treated CONs. *n* = 5–6 wells/group. **(C)** RTPCR analysis revealed that direct application of LPS to FSC media did not affect mRNA expression of neuronal *Neun* mRNA relative to vehicle-treated CONs (*n* = 5 wells/group). **(D)** Simplified schematic depicting cholinergic phenotype and lineage genes in a basal forebrain cholinergic neuron. RTPCR analyses were run in duplicate. Data are presented as mean ± SEM. *p < 0.05, ^**^p < 0.01, two-tailed *t*-tests. *Ache*, acetylcholinesterase; *Acly*, ATP citrate lyase; *Chat*, choline acetyltransferase; *Cht*, high-affinity choline transporter; *Gbx*, gastrulation brain homeobox; *Isl1*, insulin gene enhancer protein 1; *Ldb1*, LIM domain-binding protein 1; *Lhx8*, LIM/homeobox protein 8; *Ngfr*, nerve growth factor receptor; *Trka*, tropomyosin receptor kinase A; *Vacht*, vesicular acetylcholine transporter.

Cholinergic neurons form through a developmentally regulated, time lineage-specific sequence of induction of transcription factors that create and maintain the BFCN phenotype. Cholinergic neuronal phenotype is identified by expression of the ACh-synthesizing enzyme ChAT as well as numerous genes involved in the synthesis and packaging of ACh into synaptic vesicles for release. Assessment of cholinergic phenotype genes in the FSC model following direct LPS-TLR4 activation revealed decreased levels of *Acyl* (31% [±6%]; *t*(9) = 5.1, *p* < 0.01), *Chat* (59% [±6%]; *t*(9) = 3.6, *p* < 0.01), *Vacht* (58% [±3%]; *t*(5.7) = 4.7, *p* < 0.01, Welch’s *t*-test), *Ache* (43% [±3%]; *t*(9) = 4.4, *p* < 0.01), *Cht* (57% [±8%]; *t*(9) = 4.6, *p* < 0.01), and *Trka* (53% [±2%]; *t*(5.3) = 4.4, *p* < 0.01, Welch’s *t*-test) relative to vehicle-treated FSCs (Figure 2A). We also observed reduced mRNA expression of the cholinergic lineage transcription factor *Lhx8* (70% [±3%]; *t*(5.7) = 6.0, *p* < 0.01, Welch’s *t*-test) as well as other cholinergic lineage-specifying transcription factor genes (*Isl1* [62% (±5%); *t*(9) = 4.5, *p* < 0.01], *Ldb1* [37% (±4%); *t*(9) = 5.6, *p* < 0.01], *Gbx1* [67% (±6%); *t*(5.9) = 3.4, *p* < 0.05, Welch’s *t*-test], *Gbx2* [78% (±2%); *t*(5.4) = 6.5, *p* < 0.01, Welch’s *t*-test]), relative to vehicle-treated FSCs (Figure 2B). The observed reduction of cholinergic phenotype and lineage genes occur in the absence of changes in expression of the neuronal gene *Neun* (Figure 2C) similar to our NeuN + IR data. *Lhx8* is a key developmental transcription factor in BFCNs, triggering induction of other lineage-specific transcription factors and cholinergic phenotype genes (Figure 2D). However, as would be expected, LPS increased proinflammatory cytokine/chemokine mRNAs (Supplementary Figure 2A) and microglial *Iba1* and *Cdllb* while decreasing expression of some astroglial genes (Supplementary Figure 2B), consistent with LPS activation of glia. These data reveal that direct LPS-TLR4 activation in the basal forebrain decreases expression of cholinergic neuron-specific phenotype genes and cholinergic lineage transcription factors without altering NeuN expression.

### Lipopolysaccharide-TLR4 activation induces neuroimmune signaling in the basal forebrain contributing to the loss of basal forebrain cholinergic neurons

**FIGURE 3:**
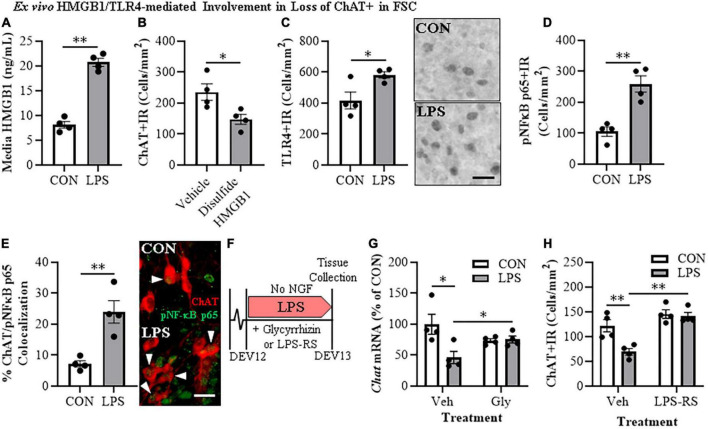
Direct TLR4 activation with LPS and disulfide HMGB1 in the FSC model induces neuroimmune signaling leading to the loss of cholinergic neurons. **(A)** ELISA assessment of HMGB1 release in basal FSC media revealed that direct application of LPS (100 ng/mL; 24 h) caused an approximate 2.5-fold increase in media levels of HMGB1 relative to vehicle-treated CONs (*n* = 4 wells/group, two-tailed *t*-test). **(B)** Modified unbiased stereology revealed that direct application of the TLR4-specific disulfide redox form of HMGB1 (0.1 μg/mL; 24 h) to FSC media caused a 37% (±7%) reduction of ChAT + IR neurons relative to vehicle-treated CONs (*n* = 4 wells/group, two-tailed *t*-test). **(C)** Modified unbiased stereology revealed that direct application of LPS to FSC media caused an approximate 1.5-fold increase of TLR4 + IR cells in the basal forebrain relative to vehicle-treated CONs. Scale bar = 50 μm (*n* = 4 wells/group, two-tailed *t*-test). **(D)** Modified unbiased stereology revealed that direct application of LPS to FSC media caused an approximate 2.5-fold increase of pNFκB p65 + IR cells in the basal forebrain relative to vehicle-treated CONs (*n* = 4 wells/group, two-tailed *t*-test). **(E)** Immunofluorescent co-labeling analysis revealed an approximate 3.3-fold increase of ChAT + IR BFCNs that co-expressed activated pNFκB p65 + IR in the basal forebrain relative to vehicle-treated CONs (*n* = 4 wells/group, two-tailed *t*-test). Representative fluorescent photomicrographs of ChAT (red) and pNFκB p65 (green) colocalization in CON- and LPS-treated FSCs. White arrow heads = ChAT + IR neuron colocalization with pNFκB p65+ (yellow). Scale bar = 50 μm. **(F)** Schematic depicting experimental design for *ex vivo* LPS HMGB1 and TLR4 blockade experiments. FSCs were treated with NGF (100 μg/mL) until day *ex vivo* (DEV) 12. On DEV12, FSCs were treated with LPS (100 ng./mL) for 24 h in the absence of NGF in combination with either the HMGB1 inhibitor glycyrrhizin (Gly; 100 μM) or the TLR4 antagonist LPS-RS (100 ng/mL) until tissue collection on DEV13. **(G)** Reverse transcription PCR analysis revealed that direct application of LPS to FSC media caused a 54% (±10%) reduction of *Chat* gene expression relative to vehicle-treated CONs. While treatment with the HMGB1 antagonist glycyrrhizin (100 μM; 24 h) alone did not affect *Chat* gene expression in CON FSC, it blunted the LPS-induced loss of *Chat* mRNA gene expression relative to LPS-treated FSCs (*n* = 4 wells/group, 2 × 2 ANOVA with follow-up two-tailed *t*-tests). RTPCR analyses run in duplicate. **(H)** Modified unbiased stereological assessment revealed that direct application of LPS to FSC media caused a 43% 5%) reduction of ChAT + IR neurons relative to vehicle-treated CONs. Although treatment with the TLR4 antagonist LPS-RS (100 ng/mL; 24 h) did not affect ChAT expression in CON FSC, it blocked the LPS-induced loss of ChAT + IR neurons (*n* = 4 wells/group, 2 × 2 ANOVA with Tukey’s HSD). Data are presented as mean ± SEM. *p < 0.05, ^**^p < 0.01.

HMGB1 is an endogenous agonist at TLR4 and is released from neurons and glia, consistent with HMGB1 activating TLR4 induction of NFκB transcription. In brain, HMGB1 is released by glutamate excitation, HDAC inhibitors, alcohol, seizure, and LPS stimulation as well as cell death (Zou and Crews, 2014, 2015; Walker et al., 2017). We assessed HMGB1 release in FSC media following LPS application and found that LPS increased media levels of HMGB1 by approximately 2.5-fold [*t*(6) = 11.9, *p* < 0.01] relative to vehicle-treated FSCs (Figure 3A). The disulfide form of HMGB1 directly binds to TLR4 (Yang et al., 2015). We applied disulfide HMGB1 (0.1 μg/mL; 24 h) to FSC and observed reduced populations of ChAT + IR BFCNs (37% [±7%]; *t*(6) = 2.9, *p* < 0.05) relative to vehicle-treated FSCs (Figure 3B) and similar to LPS (Figure 1E). TLR4 agonists are known to induce TLR4, and treatment of FSC with LPS led to an approximate 1.5-fold increase of TLR4 + IR cells [*t*(6) = 2.8, *p* < 0.05] relative to vehicle-treated FSCs (Figure 3C). Previous studies have found ChAT + IR BFCNs express TLR4 (Crews et al., 2021). HMGB1 and LPS binding to TLR4 activates NF-κB (Chavakis et al., 2004; Tobon-Velasco et al., 2014; Liu et al., 2017; Aucott et al., 2018) and transcriptionally activated pNF-κB p65 + IR is associated with increased transcription of neuroimmune genes, as has been reported in the rat basal forebrain (Vetreno and Crews, 2018; Vetreno et al., 2019; Crews et al., 2021). Application of LPS to FSC increased pNF-κB p65 + IR cells approximately 2.5-fold [*t*(3.5) = 8.4, *p* < 0.01, Welch’s *t*-test] relative to vehicle-treated FSCs (Figure 3D). Interestingly, direct TLR4 activation in the FSC model with LPS led to a 3.3-fold increase of ChAT + BFCNs that co-expressed activated pNF-κB p65 + IR [*t*(4.4) = 4.4, *p* < 0.01] relative to vehicle-treated FSCs (Figure 3E). These findings are consistent with HMGB1-TLR4-NFκB signaling within BFCNs contributing to loss of ChAT and other cholinergic neuron phenotype-specific genes.

To further examine the role of HMGB1-TLR4 neuroimmune signaling in the loss of cholinergic neurons, we assessed the effects of the HMGB1 inhibitor glycyrrhizin (100 μM) and the TLR4 antagonist LPS-RS (100 ng/mL) on FSC ChAT + BFCN LPS responses (Figure 3F). In these experiments, LPS-TLR4 activation decreased *Chat* mRNA levels 54% (±10%) [*t*(6) = 2.9, *p* < 0.05], while the HMGB1 antagonist glycyrrhizin blocked the loss of *Chat* [*t*(6) = 2.6, *p* < 0.05; Figure 3G], consistent with LPS-released HMGB1 contributing to TLR4 activation and loss of ChAT. In the TLR4 antagonist LPS-RS study, LPS alone caused a 43% (±5%) reduction of ChAT + IR neurons (Tukey’s HSD: *p* < 0.01) that was blocked by LPS-RS (Tukey’s HSD; *p* < 0.01; Figure 3H), consistent with TLR4 signaling reducing ChAT expression. Together, these findings support that HMGB1-TLR4 neuroimmune signaling, perhaps in ChAT + IR neurons as well as glia, contribute to the loss of BFCNs.

### Lipopolysaccharide-TLR4 activation in basal forebrain increases gene repression markers on key cholinergic phenotype genes

**FIGURE 4:**
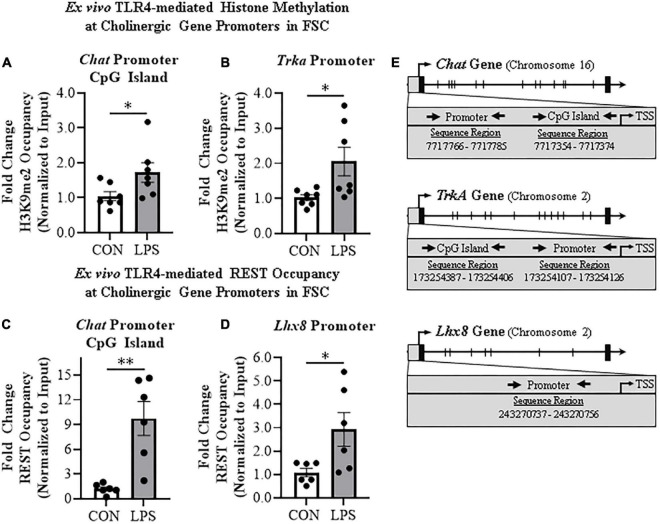
Direct TLR4 activation with LPS induces gene histone methylation and RE1 silencing transcription factor (REST) occupancy at cholinergic genes. Chromatin immunoprecipitation (ChIP) revealed that direct application of LPS (100 ng/mL; 24 h) to basal forebrain slice culture (FSC) media increased histone 3 lysine 9 dimethylation (H3K9me2) occupancy at the **(A)** CpG island in the *Chat* promoter by approximately 1.7-fold and **(B)** at the promoter of the tropomyosin receptor kinase A (*Trka*) gene by approximately 2-fold relative to vehicle-treated FSCs (*n* = 7 wells/group, two-tailed *t*-test). ChIP assessment revealed that direct application of LPS to FSC media significantly increased REST occupancy **(C)** approximately 10-fold at the *Chat* promoter CpG island and **(D)** approximately 3-fold at the promoter of the LIM/homeobox protein 8 (*Lhx8*) gene relative to vehicle-treated FSCs (*n* = 6 wells/group, two-tailed *t*-test). **(E)** Simplified schematics depicting locations of promoters and promoter CpG islands assessed for H3K9me2 and REST occupancy. Chromosome location and sequence regions for each promoter primer is presented. TSS: transcription start site. Data are presented as mean ± SEM. ChIP analyses were run in duplicate. *p < 0.05, ^**^p < 0.01.

Emerging studies find that methylation of H3K9 and the RE1-silencing transcription factor (REST) suppress gene transcription, but also regulate transcription of neuronal phenotype genes during development, likely reflecting a mechanism of neuroplasticity. We investigated epigenetic gene repressive marker occupancy at cholinergic gene promoters and promoter CpG islands found near promoter transcription start sites to provide insight into LPS-TLR4-induced gene repression (Figure 4E). We report that H3K9me2 occupancy at the CpG island located within the *Chat* gene was increased approximately 1.7-fold in LPS-treated FSCs (*t*[12] = 2.2, *p* < 0.05; Figure 4A), but no change was observed at the *Chat* promoter or *Lhx8* promoter. Similarly, LPS-TLR4 activation induced an approximate twofold increase of H3K9me2 occupancy at the *Trka* promoter (*t*[6.6] = 2.5, *p* < 0.05, Welch’s *t*-test; Figure 4B), but not at the *Trka* promoter CpG island. Thus, LPS-TLR4 activation increases H3K9me2 occupancy at the *Chat* promoter CpG island and the *Trka* promoter consistent with repression of key enzyme and NGF trophic receptor of the cholinergic neuronal phenotype.

Initially known as neuron-restrictive silencer element, REST is a key transcription gene known to suppress repressor element-1 (RE1)-containing neuronal genes in non-neuronal cells and to orchestrate development of immature circuit-integrated neurons to maturity. The *Chat* and *Lhx8* genes contain the RE1 sequence that binds REST, and REST is known to regulated the expression of *Chat* and other cholinergic genes (Shimojo and Hersh, 2004). LPS-TLR4 activation increased REST occupancy at the *Chat* promoter CpG island approximately 10-fold (*t*[5.2] = 4.1, *p* < 0.01, Welch’s *t*-test, Figure 4C). Although *Trka* does not contain the REST RE1 binding site, we did observe an approximate threefold increase of REST occupancy at the cholinergic lineage gene *Lhx8* promoter (*t*[5.6] = 2.5, *p* < 0.05, Welch’s *t*-test; Figure 4D). Thus, LPS-TLR4 activation in the basal forebrain increases REST occupancy at *Chat* and *Lhx8* promoter regions as well as H3K9me2 occupancy at promoter regions of the *Chat* and *Trka* cholinergic phenotype genes consistent with gene repression altering neuronal phenotype.

**FIGURE 5:**
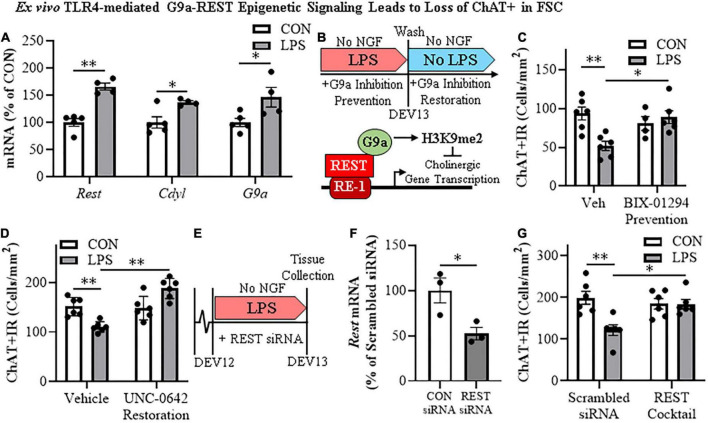
Direct TLR4 activation with LPS induces G9a-REST epigenetic signaling in the basal forebrain leading to loss of cholinergic neurons. **(A)** Reverse transcription PCR (RTPCR) revealed that direct application of LPS (100 ng/mL; 24 h) to basal forebrain slice culture (FSC) media increased gene expression of the RE1 silencing transcription factor (*Rest*) approximately 1.7-fold, the REST co-repressor *Cdyl* approximately 1.4-fold, and the methyltransferase *G9a* approximately 1.8-fold relative to vehicle-treated FSCs (*n* = 4–5 wells/group, two-tailed *t*-tests). **(B)** (TOP) Schematic depicting experimental design for *ex vivo* LPS G9a inhibitor prevention and restoration experiments. FSCs were treated with NGF (100 μg/mL) until day *ex vivo* (DEV) 12. For the prevention study, FSCs on DEV12 were treated with LPS (100 ng/mL) for 24 h in the absence of NGF in combination with either the G9a inhibitor BIX-01294 (5.0 μM) or vehicle until tissue collection on DEV13. For the restoration study, FSCs on DEV12 were treated with LPS for 24 h in the absence of NGF. Following wash to remove LPS on DEV13, FSCs were treated with either the G9a inhibitor UNC 0642 (1.0 μM) or vehicle in the absence of NGF until tissue collection on DEV14. (BOTTOM) Simplified schematic depicting the *Chat* gene containing the consensus REST repressor 21-base-pair DNA binding site RE1 (Shimojo and Hersh, 2004; Abrajano et al., 2009). REST binding to RE1 complexes with the methyltransferase G9a (Roopra et al., 2004; Ballas et al., 2005; Mulligan et al., 2008), which represses gene transcription through H3K9 dimethylation (Roopra et al., 2004; Ballas et al., 2005). **(C)** Modified unbiased stereological assessment revealed that direct application of LPS to FSC media led to a 45% (±6%) reduction of ChAT + IR neurons relative to vehicle-treated FSCs. Treatment with the G9a methyltransferase inhibitor BIX-01294 (5.0 μM) alone did not affect ChAT expression in CON FSC, whereas BIX-01294 treatment during LPS exposure blocked the loss of ChAT + IR neurons relative to LPS-treated FSCs (*n* = 4–6 wells/group, 2 × 2 ANOVA with Tukey’s HSD). **(D)** Modified unbiased stereological assessment revealed that direct application of LPS to FSC media led to a 27% (±3%) reduction of ChAT + IR neurons relative to vehicle-treated FSCs. Treatment with the G9a methyltransferase inhibitor UNC 0642 (1.0 μM) alone did not affect ChAT expression in CON FSC, but UNC 0642 treatment after LPS exposure restored the loss of ChAT + IR neurons relative to LPS-treated FSCs (*n* = 6 wells/group, 2 × 2 ANOVA with Tukey’s HSD). **(E)** Schematic depicting experimental design for *ex vivo* LPS REST siRNA blockade experiments. FSCs were treated with NGF until day *ex vivo* DEV12. On DEV12, FSCs were treated with LPS for 24 h in the absence of NGF in combination with either REST siRNA cocktail or scrambled control siRNA until tissue collection on DEV13. **(F)** RTPCR analysis revealed that administration of the REST siRNA cocktail decreased *Rest* expression 48% (±7%) in the FSC relative to scrambled CON siRNA-treated FSCs (*n* = 3 wells/group, two-tailed *t*-test). **(G)** Modified unbiased stereological assessment revealed that direct application of LPS to FSC media led to a 39% (±6%) reduction of ChAT + IR neurons relative to scrambled siRNA CON-treated FSCs. Treatment with the REST siRNA cocktail alone did not affect ChAT expression in CON FSC, but blocked the loss of ChAT + IR neurons relative to LPS-treated FSCs (*n* = 6/group, 2 × 2 ANOVA with Tukey’s HSD). RTPCR analyses run in duplicate. Data are presented as mean ± SEM. *p < 0.05, ^**^p < 0.01.

The histone methyltransferase G9a is recruited by REST, which represses gene transcription through H3K9 dimethylation (Roopra et al., 2004; Ballas et al., 2005). In our FSC model, LPS-TLR4 activation induced an approximate 1.7-fold increase of *Rest* (*t*[7] = 6.7, *p* < 0.01), a 1.4-fold increase of the REST/G9a co-repressor *Cdyl* (*t*[7] = 3.1, *p* < 0.05), and a 1.8-fold increase of *G9a* (*t*[7] = 2.6, *p* < 0.05) relative to vehicle-treated FSCs (Figure 5A). We next determined if inhibition of G9a blocks LPS-induced loss of ChAT + IR neurons in the FSC model (Figure 5B). In this experiment, LPS alone reduced ChAT + IR BFCNs by 45% (±6%; Tukey’s HSD: *p* < 0.01), and the loss of ChAT + IR was blocked by co-administration of the G9a methyltransferase inhibitor BIX-01294 (5.0 μM; Tukey’s HSD: *p* < 0.05; Figure 5C). Since these findings suggest the LPS-induced reduction of ChAT + IR involves the methyltransferase G9a, we next assessed if this loss was reversible in the FSC model (Figure 5B). FSCs were treated with LPS for 24 h as before to allow for loss of ChAT + IR; then, after LPS treatment, another G9a methyltransferase inhibitor, UNC0642 (1.0 μM), was added to cultures for 24 h following removal of LPS. We found the loss of ChAT + IR BFCNs persisted after LPS removal for 24 h, and UNC0642 restored the loss of ChAT + IR (Tukey’s HSD: *p* < 0.01; Figure 5D), consistent with reversible repression of ChAT expression and not neuronal loss. To assess the role of REST, FSCs were treated with a REST siRNA cocktail (Figure 5E) that was found to decrease *Rest* expression (Figure 5F). *Rest* knockdown with siRNA blocked the LPS-induced loss of ChAT + IR BFCNs (Tukey’s HSD: *p* < 0.05; Figure 5G). Taken together, these data support LPS-TLR4 induction of REST and the methyltransferase G9a as contributing to increases in H3K9me2 and REST occupancy at cholinergic gene promoters that repress the cholinergic phenotype.

## Discussion

**FIGURE 6:**
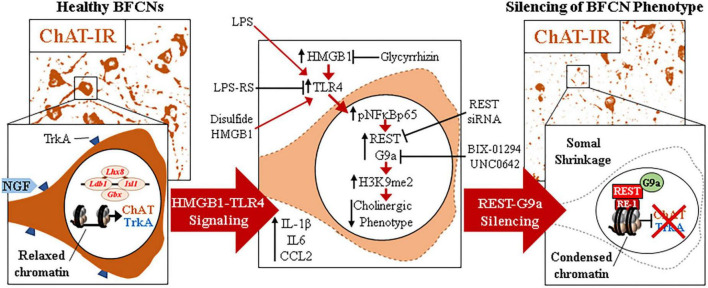
Schematic depicting the proposed neuroimmune-epigenetic mechanism underlying epigenetic repression of the cholinergic neuron phenotype. (Left) In naïve basal forebrain, healthy basal forebrain cholinergic neurons (BFCNs) express the ACh-synthesizing enzyme ChAT, the high-affinity NGF receptor tropomyosin receptor kinase A (TrkA), the LIM/homeobox protein 8 (Lhx8), and other cholinergic phenotype and lineage genes. (Top) Photomicrographs of naïve ChAT + IR neurons in the basal forebrain slice culture (FSC) model. (Bottom) Schematic depicting a ChAT + IR BFCN in orange. The *Chat* and *Lhx8* gene promoters contain the consensus 21-base-pair DNA binding sequence RE1 (Shimojo and Hersh, 2004; Abrajano et al., 2009). Note that the transcription factor RE1 silencing transcription factor (REST) is not bound to the RE1 binding site allowing relaxed chromatin and active transcription of *Chat*, *Trka*, *Lhx8*, and other cholinergic genes in healthy BFCNs. (Middle) Signaling schematic: Toll-like receptor 4 (TLR4) activation with LPS increases nuclear translocation of pNFκB p65 inducing REST, CDYL, and G9a expression as well as increased histone 3 lysine 9 dimethylation (H3K9me2) and REST occupancy at promoter regions on *Chat*, *Trka*, and *Lhx8* cholinergic genes. Inhibition of HMGB1 with glycyrrhizin, TLR4 with LPS-RS, and REST knockdown with siRNA all block LPS-induced loss of ChAT. Inhibition of the methyltransferase G9a with BIX-01294 and UNC0642 prevented and restored, respectively, the LPS-induced loss of ChAT + IR BFCNs. (Right) Neuroimmune induction causes epigenetic repression of the cholinergic phenotype. (Top) Photomicrographs of LPS-treated BFCNs depicting loss of ChAT + IR neurons and somal shrinkage of the remaining ChAT+ cholinergic neurons in the FSC model. (Bottom) Schematic depicting REST binding to RE1 sites on *Chat* and *Lhx8* genes, recruiting G9a to repress expression of *Chat*, *Trka*, and *Lhx8* in BFCNs. This condenses chromatin at cholinergic genes, repressing gene transcription through a reversible mechanism. Note that expression of the neuron-specific marker NeuN and the cell death marker propidium iodide was unchanged by LPS treatment, consistent with loss of the BFCN phenotype and not cell death. HMGB1-TLR4 signaling-induced cholinergic gene repression may represent a neuroprotective mechanism resulting in the reversible loss of highly differentiated BFCNs.

The current findings extend our *in vivo* studies reporting loss of ChAT + IR BFCNs and epigenetic repression of cholinergic genes through HMGB1-TLR4 signaling (Vetreno et al., 2014; Vetreno and Crews, 2018; Crews et al., 2021). The mammalian *Chat* gene contains transcriptional enhancers and repressors that are involved in development of BFCNs to fully mature neurons (Hersh et al., 1993; Pu et al., 1993; Hersh and Shimojo, 2003). In FSC, LPS reduced ChAT + IR BFCNs and caused somal shrinkage of the residual ChAT+ neurons without evidence of cell death or loss of the nuclear neuronal marker NeuN + IR, similar to outcomes in *in vivo* studies (Vetreno and Crews, 2018; Vetreno et al., 2019; Crews et al., 2021). In addition to reducing ChAT + IR BFCNs, LPS reduced mRNA expression of the cholinergic phenotype genes *Chat*, *Vacht*, *Acyl*, *Trka*, *Cht*, and *Ache* as well as the cholinergic lineage transcription factors *Lhx8*, *Isl1*, *Ldb1*, *Gbx1*, and *Gbx2*, but not the global neuronal nuclear marker gene *Neun*. While we observed that TLR4 neuroimmune activation with LPS did not affect protein or mRNA expression of the neuronal marker NeuN or the cell death marker propidium iodide, it is possible that some cell death occurs. However, since we find the loss of ChAT + IR BFCNs is reversed, the most parsimonious explanation is that BFCNs lose their phenotype, which can be restored. Lhx8 is a key lineage transcription factor for cholinergic neuron differentiation (Zhao et al., 2003; Mori et al., 2004), and deletion of Lhx8 in mice similarly decreases ChAT + IR BFCNs and impairs cognition without affecting total neuron populations (Fragkouli et al., 2005, 2009). LPS treatment increased expression of *Rest*, *Cdyl* and *G9a* that was accompanied by modest but significant increases in repressive H3K9me2 occupancy on *Chat* and *Trka* gene promoters and the silencing transcription factor REST on *Chat* and *Lhx8* promoter regions. REST represses genes by forming a complex with G9a and the co-repressor bridge CDYL (Roopra et al., 2004; Ballas et al., 2005; Mulligan et al., 2008). These findings suggest that LPS-TLR4 activation induces loss of ChAT + IR BFCNs through repression of *Trka*, *Chat*, and the cholinergic lineage-specifying *Lhx8* transcription factor. Both *Chat* and *Lhx8* contain the consensus REST repressor 21-base-pair DNA binding sequence RE1 (Shimojo and Hersh, 2004; Abrajano et al., 2009), indicating they are specific REST target genes in BFCNs. REST binding to RE1 complexes with the methyltransferase G9a (Roopra et al., 2004; Ballas et al., 2005; Mulligan et al., 2008), which represses gene transcription through H3K9 dimethylation (Roopra et al., 2004; Ballas et al., 2005). Lhx8 modulates expression of cholinergic phenotype through interactions with the cholinergic lineage transcription factor Isl1 (Cho et al., 2014), Gbx1/2 (Zhao et al., 2003), and the nuclear LIM domain-binding factor Ldb1 (Zhao et al., 2014) which binds enhancer regions for cholinergic genes (Cho et al., 2014). Lhx8 also activates transcription of the high-affinity NGF receptor TrkA through Lhx8 binding to the Lhx-binding sequence on the *Trka* promoter (Tomioka et al., 2014). Interaction of NGF with TrkA plays a critical role in promoting the survival and maintenance of BFCNs (Lucidi-Phillipi et al., 1996; Fagan et al., 1997). Importantly, NGF also activates transcription of Lhx8 through an Lhx8-TrkA-NGF positive feedback loop (Tomioka et al., 2014), suggesting that reductions of Lhx8 and TrkA contribute to the loss of cholinergic phenotype. Transgenic mouse studies find TLR4 neuroimmune signals alter gene repression enzymes and markers in brain (Chen et al., 2009; Montesinos et al., 2016). We found that inhibition of G9a with BIX-01294 during LPS exposure blocked the LPS-induced loss of ChAT + IR whereas treatment with the G9a inhibitor UNC0642 after removal of LPS and loss of ChAT + IR, recovered the loss of ChAT + IR BFCNs. Previous studies have reported reversible fimbria-fornix lesion-induced loss of BFCNs and somal shrinkage of remaining ChAT+ neurons (Hefti, 1986; Hagg et al., 1988, 1989) similar to our findings implicating epigenetics in the reversible loss of BFCN phenotype. Further, we found REST siRNA blocked the LPS-induced loss of ChAT + IR BFCNs. Taken together, these data suggest LPS-TLR4 activation induces REST-G9a, which represses cholinergic lineage and phenotype genes that cause shrinkage of remaining forebrain cholinergic neurons, with some losing their cholinergic phenotype but remaining neurons of an unknown, perhaps immature, phenotype (Figure 6).

The somal shrinkage and loss of ChAT phenotype in BFCNs may represent a protective mechanism or an initial phase of degeneration. REST repression of mature neuronal genes during development has been suggested to protect immature neurons, allowing for growth and development of axons and dendrites before REST is removed allowing expression of mature neuronal and synaptic genes (Zhao et al., 2017). In the adult brain, neuronal REST is low although it increases with age and stressful insults, suggesting it may be neuroprotective (Thiel et al., 2015). Human neurons show increased REST expression during normal healthy aging, but REST is not found in neurons of individuals with mild cognitive impairment and AD (Lu et al., 2014). Our *in vivo* studies, we find LPS and AIE exposure cause long-lasting increases in adult HMGB1 and TLR4 expression as well as increases in cholinergic gene repression with reduced ChAT + IR and other cholinergic phenotype genes, similar to our findings reported here in LPS-treated FSC. Further, our *in vivo* studies find treatment with indomethacin or exercise can reverse increases in TLR4 and other neuroimmune genes, and can restore loss of ChAT + IR BFCNs, which supports epigenetic gene repression mechanisms and not neuronal death (Vetreno and Crews, 2018; Vetreno et al., 2019; Crews et al., 2021). Our findings here indicate that direct LPS-TLR4 activation in FSC represses cholinergic lineage and phenotype genes, and causes somal shrinkage. Loss of ChAT+ somal size may be related to activation of neuroprotective autophagy and excretion of intracellular components. Studies of neuronal cultures undergoing “serum withdrawal,” a trophic factor loss-induced stress that induces autophagy, reveal reduced nuclear REST and increased cytosolic REST co-localized with autophagosome markers (Lu et al., 2014), consistent with autophagy reducing REST and cell size. Additional studies are needed to determine the role of autophagy in LPS-induced changes in ChAT + IR neurons with reduced soma, but reduced nuclear REST through an autophagic mechanism is consistent with cholinergic phenotype gene expression continuing in neurons of reduced size. Thus, the observed ChAT + BFCN response to LPS-TLR4 activation of increased REST, G9a, and repression of cholinergic phenotype genes as well as somal shrinkage may represent neuroprotective shrinkage with reduced expression of lineage-specific cholinergic phenotype genes.

The LPS is a TLR4 agonist that through cytosolic phosphorylation cascades activates NFκB transcription, most commonly through NFκB p50/pNF-κB p65 (i.e., RelA). Although TLRs are generally associated with microglia, TLR4 is widely expressed across brain cell types. For example, microglial depletion in brain using PLX reduces Iba-1, CD11b, CSFR1, and many other microglial genes, but does not significantly reduce brain expression of TLR4 (Walter and Crews, 2017). We report here that in FSC, LPS treatment increased pNFκB p65 + IR within ChAT + IR BFCNs. This is consistent with our previous *in vivo* finding of ChAT + IR co-expression with TLR4+ and pNFκB p65 + IR (Crews et al., 2021), suggesting that ChAT neurons have TLR4 receptors activating NFκB transcription. Neuronal NFκB activation is associated with protection from neurodegeneration and survival responses to trophic factors (Kaltschmidt and Kaltschmidt, 2009; Dresselhaus and Meffert, 2019). We report in FSC that both LPS and disulfide HMGB1, an endogenous TLR4 agonist, reduces ChAT + IR BFCNs consistent with HMGB1-TLR4 signaling through NFκB within ChAT+ neurons inducing REST and G9a, which contributes to repression of ChAT and cholinergic gene transcription. Consistent with HMGB1-TLR4 signaling contributing to the loss BFCN markers is our observation that glycyrrhizin, which inhibits HMGB1 signaling (Mollica et al., 2007) blunted whereas the TLR4 inhibitor LPS-RS blocked the LPS-induced loss of ChAT expression. LPS-TLR4 activation in FSC includes glial responses that could contribute to these effects. However, our *in vivo* findings of ChAT co-expression with TLR4 and pNFκB p65 (Crews et al., 2021) and LPS increased ChAT co-expression of pNFκB p65 in FSC are consistent with direct HMGB1-TLR4-NF-κB signaling within cholinergic neurons inducing REST and G9a that represses cholinergic phenotype genes (Figure 6).

Treatment of rats with LPS or AIE persistently increases forebrain expression of HMGB1, TLR4, and other proinflammatory genes while reducing expression of ChAT, TrkA, VAChT, and other cholinergic phenotype genes. Reductions of BFCNs in these models is paralleled by impaired behavioral flexibility as determined by reversal learning assessments using the Morris water maze (Vetreno et al., 2019) and Barnes maze (Vetreno and Crews, 2012) as well as other cognitive assessments, such as the Hebb–Williams maze series (Macht et al., 2020) and novel object recognition memory task (Vetreno and Crews, 2015). These studies and others suggest that reduced BFCN populations contribute to long-lasting cognitive deficits. Our recent findings indicate that exercise (Vetreno et al., 2019) and the anti-inflammatory drug indomethacin (Vetreno and Crews, 2018) can recover both the loss of BFCNs and cognitive deficits. We report here reversal of ChAT + IR neuron loss with the G9a inhibitor UNC0642 in the FSC model.

In summary, LPS and disulfide HMGB1 TLR4 agonists in a FSC model reduce ChAT + IR, somal size of the remaining ChAT + BFCNs, and expression of cholinergic lineage and phenotype genes without evidence of cell death or neuron loss, similar to *in vivo* studies. The transcriptional repressor *Rest*, the methyltransferase *G9a*, and the REST/G9a co-repressor *Cdyl* are induced by TLR4 activation with LPS that is accompanied by increased occupancy of repressive gene markers H3K9me2 and REST at promoter regions of the *Chat*, *Trka*, and *Lhx8*. G9a inhibitors during LPS exposure prevent, and after LPS exposure reverse, the loss of ChAT + IR BFCNs. REST siRNA knockdown similarly blocks the LPS-induced loss of ChAT. Together, these data suggest the HMGB1-TLR4 neuroimmune activation in basal forebrain reduces and shrinks cholinergic neurons through a persistent repression of cholinergic genes that is reversible. These reversible mechanisms of cholinergic neuron loss and cognitive deficits may provide new targets for treatment of cognitive impairments.

## Data availability statement

The raw data supporting the conclusions of this article will be made available by the authors, without undue reservation, to any qualified researcher.

## Ethics statement

This animal study was reviewed and approved by Institutional Animal Care and Use Committee of the University of North Carolina at Chapel Hill.

## Author contributions

RV and FC were responsible for the study concept and design and drafted the manuscript. RV was responsible for the data preparation and analysis. Both authors were involved in manuscript editing and have approved the final version for publication.
