# Supplementary material for: Cholinergic REST-G9a gene repression through HMGB1-TLR4 neuroimmune signaling regulates basal forebrain cholinergic neuron phenotype
Source: Front Mol Neurosci. 2022 Aug 22;15:992627. doi: 10.3389/fnmol.2022.992627 (PMC9441808; doi:10.3389/fnmol.2022.992627)
Supplement: Supplementary file 1 [file Data_Sheet_1.docx]

**
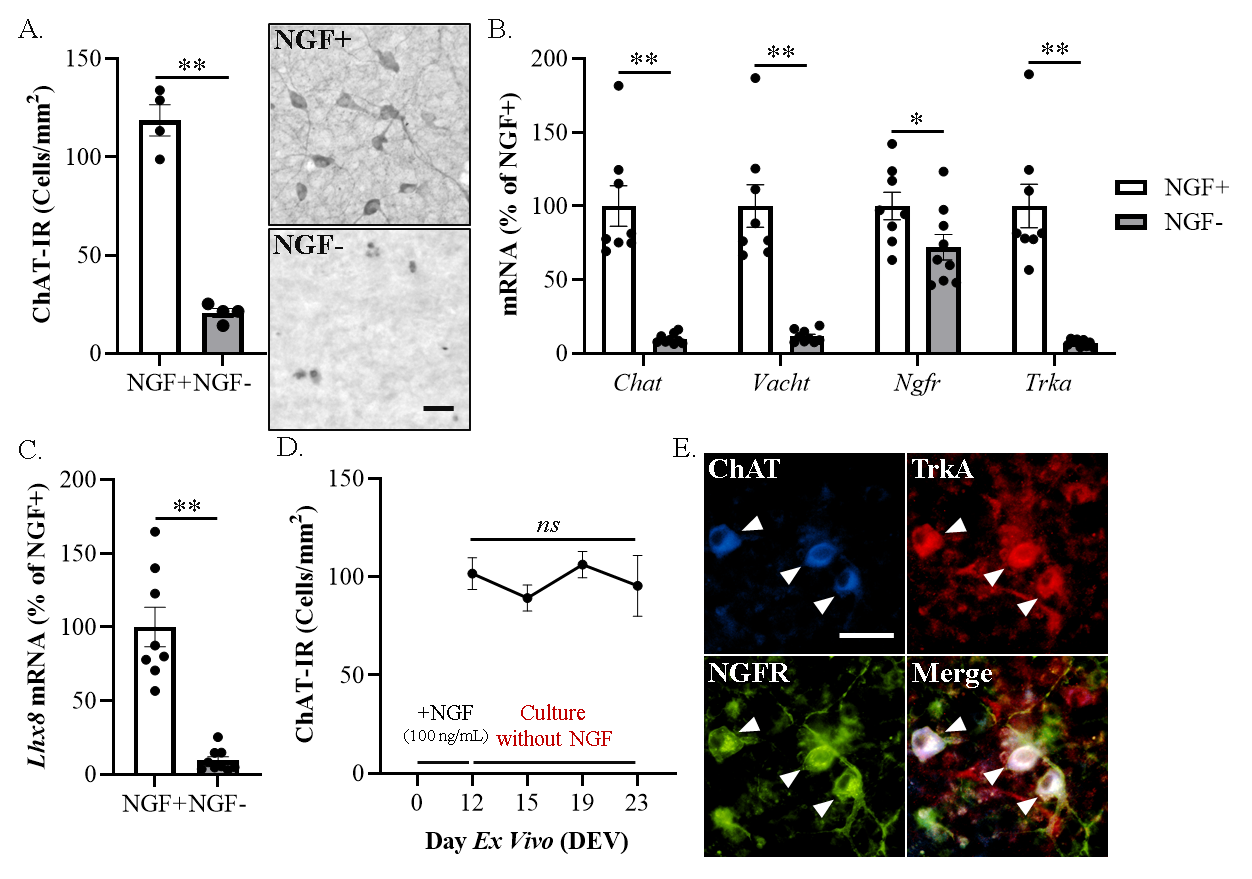
Supplemental Figure 1. Exclusion of NGF during experimental manipulation does not affect ChAT+IR in the basal forebrain slice culture (FSC) model.** (A) Immediately following dissection, FSCs were grown in media containing either NGF (NGF+; 100 ng/mL) or no NGF (NGF-) for 12 days *ex vivo* (DEV) (n = 4 wells/group). At 12 DEV in the absence of NGF, we found an 83% (±2%) reduction of ChAT+IR neurons relative to NGF+ CONs (*t*[6]=11.8, *p*<0.01, two-tailed *t* test). Representative photomicrographs of ChAT+IR neurons in FSC grown in media containing NGF or no NGF. Note that deprivation of NGF during the initial growth of basal forebrain cholinergic neurons (BFCNs) robustly decreased the number and size of BFCNs. Scale bar = 50 μm. In an additional cohort, basal forebrain sections that were collected on P8 (0 DEV) contained 251 (±31; n = 3) ChAT+IR neurons. These data reveal a critical role of NGF in the development and maturation of BFCNs, whereas mature BFCNs can survive up to two weeks *ex vivo* in the absence of NGF (Weis et al., 2001). (B) Reverse transcription PCR (RTPCR) revealed that NGF deprivation (i.e., NGF-) in the FSC for 12 DEV decreased mRNA expression of cholinergic phenotype genes (*Chat* [90% (±1%); *t*(7.1)=6.6, *p*<0.01, Welch’s *t* test], *Vacht* [89% (±1%); *t*(7.1)=6.1, *p*<0.01, Welch’s *t* test], *Ngfr* [28% (±9%); *t*(15)=2.2, *p*<0.05], *Trka* [93% (±0%)]; *t*(7.0)=6.3, *p*<0.01, Welch’s *t* test) and (C) the cholinergic lineage gene *Lhx8* (91% [±3%]; *t*(7.5)=6.6, *p*<0.01, Welch’s *t* test) relative to NGF+ CONs (n = 8-9 wells/group, two-tailed *t* tests). RTPCR analyses run in duplicate. (D) At the beginning of experimentation, FSCs are treated with NGF (100 ng/mL) in media for 12 DEV based on our protocol followed by culture in NGF-deprived media to 23 DEV to determine survival of ChAT+IR BFCNs. Modified unbiased stereological assessment revealed that NGF deprivation did not affect ChAT+IR neurons (n = 3 wells/group). (E) Immunofluorescent co-labeling revealed a high degree of tropomyosin receptor kinase A (TrkA [red]) and nerve growth factor receptor (NGFR [green]) colocalization with ChAT+IR BFCNs (blue) in the FSC model. Scale bar = 50 μm. Data are presented as mean ± SEM. * *p*<0.05, ** *p*<0.01.

**Supplemental Figure 2.** **Direct TLR4 activation with LPS in the FSC model induces proinflammatory cytokine and chemokines genes as well as microglial genes in the basal forebrain.** (A) Reverse transcription PCR (RTPCR) revealed that direct application of LPS (100 ng/mL; 24 h) to basal forebrain slice culture (FSC) media increased gene expression of the proinflammatory cytokines *Tnfα* (3.2-fold; *t*(4.4)=6.2, *p*<0.01, Welch’s *t* test), *Il1β* (18-fold; *t*(4.0)=7.5, *p*<0.01, Welch’s *t* test), and *Il6* (29-fold: *t*(4.0)=8.6, *p*<0.01, Welch’s *t* test) as well as the chemokine *Ccl2* (236-fold; *t*(4.0)=6.1, *p*<0.01, Welch’s *t* test) relative to vehicle-treated FSC. (B) RTPCR analysis revealed that LPS treatment of FSC media increased expression of microglial genes *Iba1* (3-fold; *t*(8)=10.5, *p*<0.01) and *Cd11b* (2.5-fold; *t*(8)=4.0, *p*<0.01), and decreased expression of astrocytic genes *Gfap* (68% [±4%]; *t*(8)=4.7, *p*<0.01), *Gs* (37% [±8%]; *t*(8)=3.8, *p*<0.01), and *S100b* (70% [±4%]; *t*(8)=4.4, *p*<0.01), but not *Glt1*, relative to vehicle-treated FSCs (n = 4-5 wells/group, two-tailed *t* test). RTPCR analyses run in duplicate. Data are presented as mean ± SEM. * *p*<0.05, ** *p*<0.01
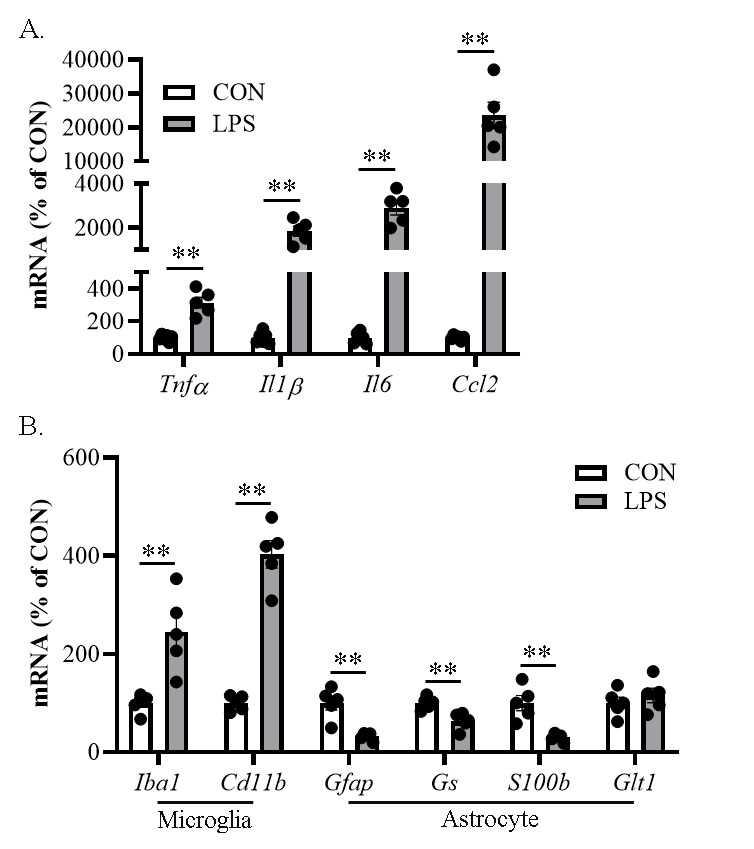
. *Ccl2*: chemokine (C-C motif) ligand 2; *Cd11b*: cluster of differentiation molecule 11b; *Gfap*: glial fibrillary acidic protein; *Glt1*: glutamate transporter 1; *Gs*: glutamine synthetase; *Iba1*: ionized calcium-binding adapter molecule 1; *Il1β*: interleukin 1 beta; *Il6*: interleukin 6; *S100b*: S100 calcium-binding protein B; *Tnfα*: tumor necrosis factor alpha.
